# Anatomical localization of normal parathyroid glands before thyroidectomy through ultrasonography reduces postoperative hypoparathyroidism

**DOI:** 10.1097/MD.0000000000016020

**Published:** 2019-06-14

**Authors:** Jin-Duo Shou, Shui-Ming He, Xian-Feng Jiang, Liu-Hong Shi, Lei Xie, Jian-Biao Wang

**Affiliations:** a Department of Diagnostic Ultrasound and Echocardiography; b Department of Head and Neck Surgery, Sir Run Run Shaw Hospital, Zhejiang University School of Medicine, Hangzhou, Zhejiang; c Department of Oral and Maxillofacial Surgery, Shaoxing Central Hospital, Shaoxing, Zhejiang, China.

**Keywords:** anatomical localization, hypoparathyroidism, parathyroid gland, thyroidectomy, ultrasonography

## Abstract

Postoperative hypoparathyroidism due to dysfunction of the parathyroid gland is the most common complication after thyroidectomy. Our objective was to introduce the method of anatomical localization of normal parathyroid glands before thyroidectomy through ultrasonography and to evaluate its efficiency. The study group included 52 patients subjected to anatomical localization of the parathyroid gland prethyroidectomy through ultrasonography. The control group included 52 sex- and age-matched patients without parathyroid gland localization. The proportion of parathyroid glands preserved in situ and postoperative hypoparathyroidism rates in the 2 groups were compared. The rates of normal parathyroid glands identified according to ultrasonography for left superior, left inferior, right superior, and right inferior glands were 78.8%, 90.4%, 57.7%, and 82.7%, respectively. The rate of parathyroid gland excised inadvertently was significantly decreased (*P* = .038) in the study group as compared with the control group. The rates of parathyroid gland preservation in situ were significantly improved in the left superior (*P* = .001), left inferior (*P* = .002), and right inferior glands (*P* = .005). Furthermore, the incidence of transient hypoparathyroidism decreased significantly (*P* = .028). Our study indicated that normal parathyroid glands were identified by ultrasonography, and the anatomical localization improved the rate of parathyroid gland preservation in situ and decreased the incidence of transient hypoparathyroidism.

## 1 Introduction

Postoperative hypoparathyroidism is the most common complication of thyroidectomy and leads to prolonged hospitalization and medical care.^[1–4]^ The prevalence rates of transient and permanent hypoparathyroidism range from 14% to 60% and from 4% to 11%, respectively.^[5]^ The most common reasons for hypoparathyroidism include mechanical damage, devascularization, or inadvertent removal of the parathyroid gland.^[6,7]^ A recent study by Lorente-Poch et al demonstrated that the more numbers of parathyroid glands preserved in situ, the less incidence of transient and permanent hypocalcemia. The study suggested that the parathyroid glands are identified and preserved in situ, and autotransplantation should be limited to those accidentally devascularized glands that cannot be preserved.^[8]^ To date, various studies have focused on the early detection of hypocalcemia or replacement with calcium supplementation. However, an exhaustive literature search retrieved few formal data regarding methods for in situ preservation of parathyroid gland, thereby reducing the incidence of hypoparathyroidism.

The present study hypothesized that identifying the anatomical position of the normal parathyroid gland and the correlation between the thyroid lobe, common carotid artery (innominate artery), trachea, esophagus, and thyroid vein before the operation could facilitate the detection, identification, and the in situ preservation of parathyroid gland during thyroidectomy, thereby decreasing the incidence of hypoparathyroidism. Thus, this study aimed to introduce the method of anatomical localization of normal parathyroid glands before thyroidectomy through ultrasonography and to evaluate its effects on hypoparathyroidism in patients, who underwent thyroidectomy and central neck dissection (CND).

## 2 Materials and methods

### 2.1 Patients

Patients with thyroid carcinoma or nodular goiter, who underwent thyroidectomy or CND between May 1, 2017 and July 31, 2018, were reviewed in a retrospective study. The exclusion criteria were as follows: previous thyroidectomy or parathyroidectomy, preoperative hypocalcaemia, surgical extent less than thyroid lobectomy, and osteoporosis requiring treatment with calcium or vitamin D.

The identification of the anatomical position of normal parathyroid glands by ultrasonography before thyroidectomy was first introduced by Jinduo Shou in May 2017, and the patients who were subjected to this procedure were enrolled in the present study. The cohort consisted of 52 patients (47 women and 5 men) with mean age 46.8 years (range, 23–70 years). This study group was compared to a control group of sex- and age-matched patients who did not undergo ultrasonography of the parathyroid gland between May 1, 2017 and July 31, 2018 (mean age, 46.7 years; range, 20–75 years; 46 women and 6 men).

The present study protocol was approved by the Ethics Committee of Sir Run Run Shaw Hospital, School of Medicine, Zhejiang University.

### 2.2 Anatomical localization of normal parathyroid glands through ultrasonography

We used EPIQ 5 iU22 (Philips, The Netherlands) or LOGIC E8 (GE, USA) machines equipped with L523 (5–15 MHz) linear transducer for ultrasonography examination of normal parathyroid glands before thyroidectomy. The conventional gray-scale and power-Doppler ultrasounds were used to search and identify the parathyroid gland during ultrasonography. All examinations were performed by the same investigator with 10 years of experience in thyroid ultrasound. The patients were placed in a supine position with the neck slightly extended by a roll beneath the shoulders.

**Figure 1:**
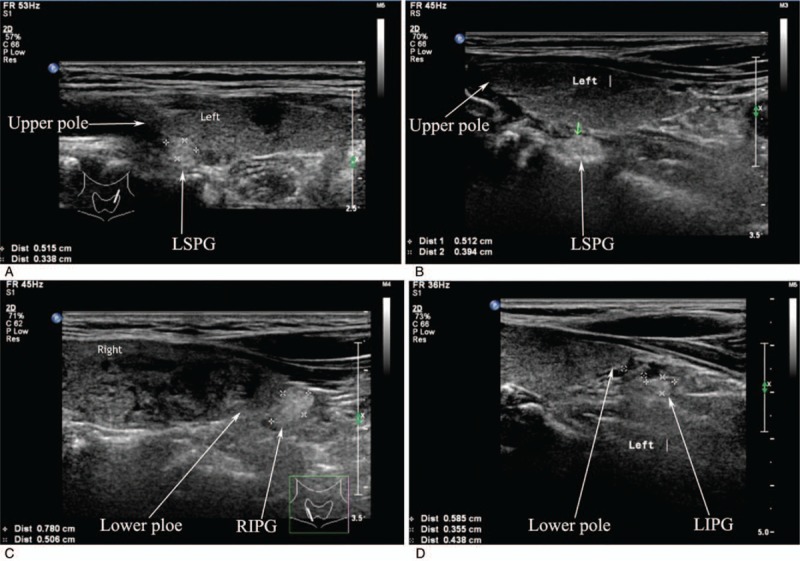
Longitudinal location of the normal parathyroid glands. (A) Ultrasonographic appearance of a LSPG located on the dorsal part of the upper third thyroid lobe; (B) ultrasonographic appearance of a LSPG located on the dorsal part of the middle third thyroid lobe; (C) Ultrasonographic appearance of a RIPG located on the dorsal part of the lower third thyroid lobe; (D) Ultrasonographic appearance of a LIPG placed 0.438 cm below the lower thyroid lobe. Longitudinal scan: LIPG = left inferior parathyroid gland; LSPG = left superior parathyroid gland; RIPG = right inferior parathyroid gland.

**Figure 2:**
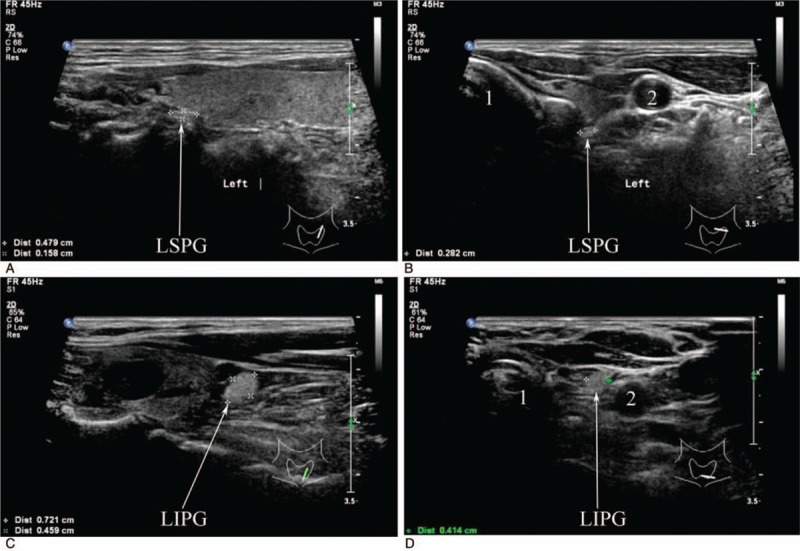
Transverse location of the normal parathyroid glands. (A) (longitudinal scan) and (B) (transverse scan), ultrasonographic appearance of a LSPG located on the dorsal part of the upper third thyroid lobe and in proximity to the trachea; (C) (longitudinal scan) and (D) (transverse scan), ultrasonographic appearance of a LIPG placed below the lower thyroid lobe and close to the common carotid artery. LIPG = left inferior parathyroid gland; LSPG = left superior parathyroid gland.

**Figure 3:**
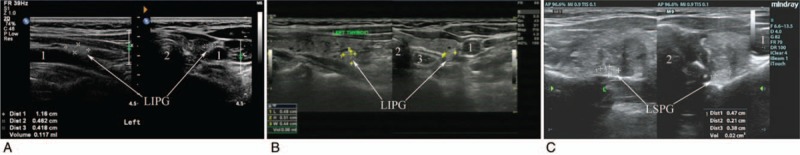
The layers of the normal parathyroid glands. (A) Ultrasonographic appearance of a LIPG located above the common carotid artery; (B) ultrasonographic appearance of a LIPG located close to the posterior capsule of the lower third thyroid lobe and near the esophagus; (C) ultrasonographic appearance of a LSPG embedded into the thyroid lobe and attached to the thyroid tumor. LIPG = left inferior parathyroid gland; LSPG = left superior parathyroid gland; 1 = common carotid artery; 2 = trachea; 3 = esophagus.

The thyroid lobe was selected as the reference, and the longitudinal positions of the parathyroid gland were divided into cranial to the upper thyroid lobe, dorsal to the upper third thyroid lobe, dorsal to the middle third thyroid lobe, dorsal to the lower third thyroid lobe, and caudal to the lower thyroid lobe (Fig. 1). Next, the carotid artery and the trachea were selected as the reference; the transverse positions of the parathyroid gland were divided as close to the trachea, close to the common carotid artery, and intermediate of the trachea and common carotid artery (Fig. 2). The localized layers of the parathyroid gland were classified as embedded into the thyroid lobe, planar attached to the thyroid lobe, attached to the esophagus, and beneath or above the common carotid artery or innominate artery (Fig. 3).

### 2.3 Surgical approach for thyroidectomy and CND

All the procedures involved were conventional open surgeries and performed by 1 senior surgeon or 2 surgical fellows under his direct supervision. In the study group, the surgeon established the anatomical position of the parathyroid glands and the correlations between the thyroid lobe, common carotid artery (innominate artery), trachea, esophagus, and thyroid vein as provided by ultrasonography preoperatively. In the control group, the surgeon lacked any information about the position of the parathyroid glands before surgery.

Thyroidectomy was performed using a standard technique of meticulous capsular dissection. The recurrent laryngeal nerves and all parathyroid glands were routinely identified and preserved under direct vision. The CND was conducted strictly according to the American Thyroid Association (ATA) guidelines.^[9]^ Bilateral CND involved the removal of prelaryngeal, pretracheal, and both the right and left paratracheal nodal basins, whereas the unilateral CND involved the removal of the prelaryngeal, pretracheal, and one paratracheal nodal basin. During thyroidectomy and CND, the parathyroid gland was searched and preserved. After thyroidectomy and CND, the parathyroid gland was identified, and the blood supply was determined by the fine-needle pricking test. A gland with oozing blood after the test was considered to be vascularized, whereas otherwise devascularized. Moreover, any devascularized parathyroid gland was removed and autotransplanted into the sternocleidomastoid muscle.

### 2.4 Clinical parameters

The data were collected on parameters including sex, age, Hashimoto's thyroiditis, body mass index (BMI), voice change, maximum tumor size, extrathyroidal extension, multifocality, number of harvested and metastatic lymph nodes, number of parathyroid glands identified during thyroidectomy, and number of parathyroid glands preserved in situ, autotransplanted, and inadvertently excised. The number of parathyroid glands excised inadvertently was examined based on the paraffin-embedded specimens of thyroid lobe and the fibrofatty tissue of the central compartment.

### 2.5 Definition and treatment of hypoparathyroidism

Transient hypoparathyroidism was defined as a subnormal level of serum intact parathyroid hormone (iPTH) (normal range 15–65 ng/L), serum calcium level <2 mmol/L (8 mg/dL), or requirement of calcium supplement to treat the clinical symptoms of hypocalcaemia, such as distal digital paraesthesia or tetany during the hospital stay.^[8,10]^ Permanent hypoparathyroidism was defined as a subnormal serum iPTH level, calcium level <2 mmol/L, or requirement of calcium and/or vitamin D supplement to treat hypocalcaemia-related symptoms for >6 months. The calcium supplements containing 750 mg calcium carbonate plus 60 units vitamin D3 were administered 2 times daily to treat the severe symptomatic hypocalcaemia.

### 2.6 Statistical analysis

Continuous data are presented as mean (SD) or median (range). The baseline patient characteristics, the incidence of hypoparathyroidism, the proportion of parathyroid glands identified, and the in situ preservations were compared between the study and control groups using the Student *t* test or Mann–Whitney *U* test for continuous variables and Pearson χ^2^ test for categorical variables. *P*<.05 was considered statistically significant. Data were analyzed using SPSS version 16.0 (IBM, Armonk, NY).

## 3 Results

### 3.1 Patient characteristics

A total of 104 patients were eligible for the present study. The male:female ratio was 11:93 (1: 8.5), and the mean age was 46.8 years. The study group consisted of 52 patients, including 47 patients with thyroid carcinoma (46 cases of papillary thyroid carcinoma [PTC] and 1 case medullary thyroid carcinoma) and 5 patients with nodular goiter, and the control group consisted of 52 patients, including 48 patients with PTC and 4 patients with nodular goiter. Parameters such as age, sex, the prevalence of Hashimoto's thyroiditis, BMI, tumor size, rates of multifocal malignant disease and extrathyroidal extension, and the number of harvested and metastatic lymph nodes did not differ significantly between the study and control groups (Table 1). The transient vocal cord palsy occurred in 1 patient in the study group, and permanent vocal cord palsy occurred in 1 patient in the control group, which was caused by the intraoperative resection of the recurrent laryngeal nerve infiltrated by tumor.

**Table 1:**
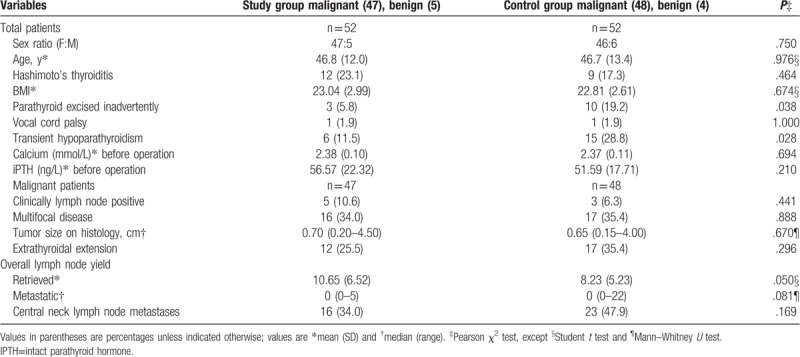
Baseline characteristics of 104 patients who underwent thyroidectomy.

### 3.2 Anatomical localization of normal parathyroid glands before thyroidectomy through ultrasonography

In the study group, 52 patients underwent ultrasonography examination for the anatomical localization of the normal parathyroid glands before surgery. The superior parathyroid gland was identified in 41 patients (78.8%) on the left side and in 30 patients (57.7%) on the right side, respectively. In addition, the inferior parathyroid gland was identified in 47 patients (90.4%) on the left side and in 43 patients (82.7%) on the right side, respectively (Table 2). The size of the parathyroid gland and the positional correlation between the parathyroid gland and thyroid gland were both measured by ultrasonography (Table 2). The size of the parathyroid gland (the craniocaudal dimension by the transverse dimension) was (0.58 ± 0.14) × (0.34 ± 0.10) cm for the left superior gland, (0.61 ± 0.20) × (0.38 ± 0.13) cm for the left inferior gland, (0.59 ± 0.17) × (0.35 ± 0.10) cm for the right superior gland, and (0.59 ± 0.19) × (0.37 ± 0.09) cm for the right inferior gland.

**Table 2:**
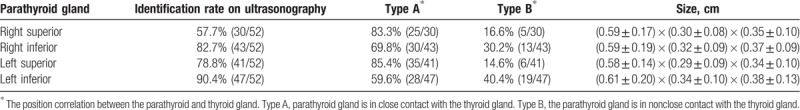
Identification rate and baseline data of parathyroid glands on ultrasonography.

### 3.3 Identification and protection of parathyroid glands during thyroidectomy

The anatomical localization of the parathyroid gland before thyroidectomy would facilitate the detection, identification, and the in situ preservation of the parathyroid glands during thyroidectomy. The rate of parathyroid glands identified by the surgeon intraoperatively in the study group was significantly higher than that in the control group (left superior parathyroid gland: 100% vs 80%, *P* = .004) (Table 3). Moreover, the rate of inadvertently excised parathyroid was significantly lower in the study group than that in the control group (5.8% vs 19.2%, *P* = .038) (Table 1).

**Table 3:**
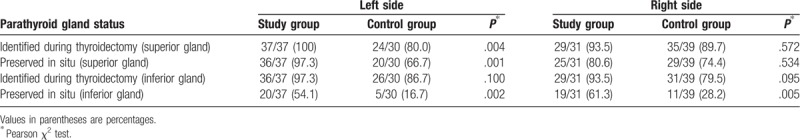
Status of parathyroid gland during thyroidectomy.

The rates of parathyroid glands preservation in situ were significantly higher in the study group as compared with the control group (superior parathyroid gland on the left side: 97.3% vs 66.7%, *P* = .001; inferior parathyroid gland on the left side: 54.1% vs 16.7%, *P* = .002; inferior parathyroid gland on the right side: 61.3% vs 28.2%, *P* = .005) (Table 3).

In the study group, 6/52 patients (11.5%) presented transient hypoparathyroidism as compared to 15/52 patients (28.8%) in the control group (*P* = .028). However, permanent hypoparathyroidism was not observed in either of the groups.

## 4 Discussion

The in situ preservation of parathyroid glands is the optimal method for avoiding postoperative hypoparathyroidism. During thyroidectomy and CND, typically, the parathyroid gland (especially inferior gland) undergoes dissection 2 times. It is first exposed and preserved by a meticulous capsular dissection during thyroid lobectomy, followed by identification and preservation in situ, whereas the central neck fibrofatty tissue is removed along with the lymph nodes. Failure to identify the parathyroid gland during thyroid lobectomy makes the in situ preservation in CND difficult owing to the variable positions of the glands in the adult neck ^[11]^ and the area of central neck lymph node dissection. However, if the anatomical position of the parathyroid gland is distinct before the operation, then searching and identifying the parathyroid gland during thyroidectomy and CND is easy. Therefore, when the parathyroid gland is identified, the in situ preservation is easy.

The ultrasonography in the present study displayed the normal parathyroid glands and the positional correlations between the thyroid lobe, common carotid artery (innominate artery), trachea, and esophagus. The rates of normal parathyroid glands displayed on ultrasonography for left superior, left inferior, right superior, and right inferior glands were 78.8%, 90.4%, 57.7%, and 82.7%%, respectively.

Herein, we established the method of anatomical localization of normal parathyroid glands through ultrasonography. The findings showed that anatomical localization of parathyroid gland before thyroidectomy increased the rate of identification and preservation in situ during thyroidectomy, and decreased the incidence of parathyroid gland excised inadvertently as well as the prevalence of transient hypoparathyroidism.

Furthermore, the ultrasonography examination of the parathyroid gland before thyroidectomy revealed the longitudinal position of the parathyroid gland in reference to the thyroid gland lobe, the transverse position of the gland in reference to the common carotid artery and the trachea, and the located layer of the gland in reference to the thyroid lobe, esophagus, and common carotid artery. Strikingly, the insight into the three-dimensional position of the parathyroid glands would assist the surgeons in detecting and identifying the glands during thyroid surgery.

According to the positional correlation between the parathyroid gland and thyroid gland, Zhu et al^[12]^ classified the parathyroid gland into type A (close contact) and type B (nonclose contact). Type A included A1 (planar attachment), A2 (embedded attachment), and A3 (intrathyroid), whereas type B comprised B1 (around the thyroid), B2 (intrathymus), and B3 (blood supply from thymus or mediastinum). The ultrasonography examination of the parathyroid gland revealed the type of the gland before surgery. In this study, we found that the proportion of type A according to ultrasonography was 85.4% for the left superior gland, 59.6% for the left inferior gland, 83.3% for the right superior gland, and 69.8% for the right inferior gland, respectively (Table 2).

When the type of the parathyroid gland is clarified before the operation, surgeons can exert caution during the procedure in order to preserve the parathyroid gland in situ. The thyroid lobectomy should be performed extremely carefully in type A parathyroid glands (especially A2) to preserve the parathyroid gland closely attached to the thyroid gland. In the case of the parathyroid gland of type B, surgeons should focus on CND in order to preserve the gland in situ.

Despite careful dissection and familiarity with anatomy, the parathyroid tissue is frequently removed from the neck inadvertently during thyroid operations. Reportedly, the incidental parathyroidectomy occurs in 5.2% to 21.6% of the thyroid operations.^[13–15]^ Paek et al reported that the parathyroid gland excised inadvertently was a major risk factor for both postoperative transient and permanent hypoparathyroidism.^[7]^ The study demonstrated that the frequency of such removal of the parathyroid gland was 29%.^[7]^ Recently, Applewhite et al found that patients with incidental parathyroidectomy exhibited postoperative biochemical and symptomatic hypocalcemia than the controls, and the number of parathyroid glands identified intraoperatively was inversely correlated with the number of parathyroid glands in the specimen.^[16]^ In the present study, we observed that the anatomical localization of normal parathyroid glands before operation significantly increased the rate of parathyroid glands identified intraoperatively, thereby decreasing the rate of parathyroid gland excised inadvertently (from 19.2% to 5.8%; *P* = .038).

In 2016, Dorazi et al reported the incidences of transient and permanent hypoparathyroidism were 10.7% (84 of 782 patients) and 0.38% (3 of 782 patients), respectively, with the use of loupes magnification and microsurgical technique in thyroid surgery. Dorazi et al suggested the use of loupes magnification and microsurgical technique as a common tool to visualize and preserve the parathyroid glands in thyroid surgery. However, Dorazi's study did not have a control group of patients undergoing standard thyroid surgery.^[17]^ So, it is hard to confirm whether the low incidence of hypoparathyroidism was related to the application of microsurgical technique and loupes or just own to the author's excellent surgical experience.

As is known, technetium (Tc-99^m^)-sestaMIBI is used as radiotracer for intraoperative localization of parathyroid adenomas and the injection is usually performed 2 to 3 hours before surgery because Tc-99^m^-sestaMIBI is washed out more quickly from the normal thyroid and parathyroid tissue than from the parathyroid adenomas and hyperplastic glands.^[18,19]^ Recently, Pasta et al further developed this technology through inhibit the interference of uptake of the thyroid gland by means of the administration of 10% Lugol's solution, which is useful both to the certain identification and intraoperative localization of the parathyroid adenoma by gamma probe.^[20]^ However, up to now, Tc-99^m^-sestaMIBI is only used for the localization of parathyroid adenomas, whereas normal parathyroid glands still canot be detected by Tc-99^m^-sestaMIBI scintigraphy.

Although several exogenous dyes (methylene blue and indocyanine green) have been used to localize the parathyroid glands intraoperatively, IV injection is essential that might lead to symptoms such as neurotoxicity, photobleaching, and pain at the infusion site.^[21–23]^ Moreover, fluorescence images of the parathyroid tissue cannot be acquired when the glands are covered with fat, vessels, connective tissues, or thyroid gland. This means that the putative location of the parathyroid gland is not identified until the gland is exposed. Thus, these methods are not valuable to the operator in detecting and identifying the parathyroid gland during thyroidectomy and CND.

Nevertheless, the present study had some limitations, such as the limited number of enrolled patients, the retrospective design of the study, and the nonconsecutive collection of the patients. In addition, the control group in this study was only age- and sex-matched but not randomized. Although the baseline characteristics and TNM stage did not exhibit significant differences between the study and control groups, selective biases may be observed. Thus, the usefulness of the anatomical localization technique of normal parathyroid glands before thyroidectomy to prevent hypoparathyroidism needs further substantiation by randomized control trials.

In conclusion, we developed a method to identify and anatomically localize the normal parathyroid glands before thyroidectomy using ultrasonography examination. This technique of anatomical localization preoperatively can aid the surgeons in preserving the parathyroid glands in situ during thyroidectomy and CND, thereby reducing the transient hypoparathyroidism postthyroidectomy.

## Author contributions

**Conceptualization:** Jin-Duo Shou, Jian-Biao Wang.

**Data curation:** Jin-Duo Shou, Shui-Ming He, Xian-Feng Jiang, Liu-Hong Shi.

**Formal analysis:** Shui-Ming He, Jian-Biao Wang.

**Funding acquisition:** Jian-Biao Wang.

**Investigation:** Jin-Duo Shou, Shui-Ming He, Xian-Feng Jiang, Liu-Hong Shi, Lei Xie, Jian-Biao Wang.

**Methodology:** Jin-Duo Shou, Shui-Ming He, Xian-Feng Jiang, Liu-Hong Shi, Lei Xie, Jian-Biao Wang.

**Supervision:** Jian-Biao Wang.

**Writing – original draft:** Jin-Duo Shou, Shui-Ming He, Lei Xie, Jian-Biao Wang.

**Writing – review and editing:** Jian-Biao Wang.
